# Fully Automated Wound Tissue Segmentation Using Deep Learning on Mobile Devices: Cohort Study

**DOI:** 10.2196/36977

**Published:** 2022-04-22

**Authors:** Dhanesh Ramachandram, Jose Luis Ramirez-GarciaLuna, Robert D J Fraser, Mario Aurelio Martínez-Jiménez, Jesus E Arriaga-Caballero, Justin Allport

**Affiliations:** 1 Swift Medical Inc Toronto, ON Canada; 2 Department of Surgery Universidad Autonoma de San Luis Potosi San Luis Potosi Mexico

**Keywords:** wound, tissue segmentation, automated tissue identification, deep learning, mobile imaging, mobile phone

## Abstract

**Background:**

Composition of tissue types within a wound is a useful indicator of its healing progression. Tissue composition is clinically used in wound healing tools (eg, Bates-Jensen Wound Assessment Tool) to assess risk and recommend treatment. However, wound tissue identification and the estimation of their relative composition is highly subjective. Consequently, incorrect assessments could be reported, leading to downstream impacts including inappropriate dressing selection, failure to identify wounds at risk of not healing, or failure to make appropriate referrals to specialists.

**Objective:**

This study aimed to measure inter- and intrarater variability in manual tissue segmentation and quantification among a cohort of wound care clinicians and determine if an objective assessment of tissue types (ie, size and amount) can be achieved using deep neural networks.

**Methods:**

A data set of 58 anonymized wound images of various types of chronic wounds from Swift Medical’s Wound Database was used to conduct the inter- and intrarater agreement study. The data set was split into 3 subsets with 50% overlap between subsets to measure intrarater agreement. In this study, 4 different tissue types (epithelial, granulation, slough, and eschar) within the wound bed were independently labeled by the 5 wound clinicians at 1-week intervals using a browser-based image annotation tool. In addition, 2 deep convolutional neural network architectures were developed for wound segmentation and tissue segmentation and were used in sequence in the workflow. These models were trained using 465,187 and 17,000 image-label pairs, respectively. This is the largest and most diverse reported data set used for training deep learning models for wound and wound tissue segmentation. The resulting models offer robust performance in diverse imaging conditions, are unbiased toward skin tones, and could execute in near real time on mobile devices.

**Results:**

A poor to moderate interrater agreement in identifying tissue types in chronic wound images was reported. A very poor Krippendorff α value of .014 for interrater variability when identifying epithelization was observed, whereas granulation was most consistently identified by the clinicians. The intrarater intraclass correlation (3,1), however, indicates that raters were relatively consistent when labeling the same image multiple times over a period. Our deep learning models achieved a mean intersection over union of 0.8644 and 0.7192 for wound and tissue segmentation, respectively. A cohort of wound clinicians, by consensus, rated 91% (53/58) of the tissue segmentation results to be between fair and good in terms of tissue identification and segmentation quality.

**Conclusions:**

The interrater agreement study validates that clinicians exhibit considerable variability when identifying and visually estimating wound tissue proportion. The proposed deep learning technique provides objective tissue identification and measurements to assist clinicians in documenting the wound more accurately and could have a significant impact on wound care when deployed at scale.

## Introduction

### Overview

Wounds result from the breakdown in the protective function of the skin and the loss of continuity of the epithelium. Wounds can be generally categorized into acute and chronic wounds. Normal wound healing involves four overlapping stages: hemostasis, inflammation, proliferation, and remodeling. Wound closure can be observed between several weeks to several months depending on wound size and other patient factors. Although debatable, generally wounds taking >3 months to heal are considered chronic wounds [1]. Wound progress through the 4 phases of healing is generally assessed using subjective observation of changes in size and tissue types by clinicians. Improvement to these subjective measures offers potential for better assessment of healing, improved treatment selection, and potential to predict patients at risk of developing a chronic or nonhealing wound.

Estimates indicate that 40 million patients worldwide may be affected by chronic wounds. A recent study that examined the prevalence of wounds in Canada between 2011 and 2012 found that almost 4% of inpatient acute hospitalization clients, >7% of home-care clients, almost 10% of long-term care clients, and almost 30% of hospital-based continuing care clients developed compromised wounds. Chronic wound care also imposes a hefty economic burden on the national health care system. The adverse economic impact of wound care has been well studied [2,3]. For example, it is estimated that the total direct-care cost of diabetic foot ulcers to the Canadian health care system was determined to be CAD $547 million (US $546.6 million), with an average cost per case of CAD $21,371 (US $21,364). A major concern is to ensure that health care professionals provide timely and effective wound care to affected individuals. Although better treatment protocols, drugs, and tissue regeneration methods are being constantly developed, it is imperative that research into timely treatment and wound healing monitoring is pursued in parallel. The protocols for treatments and medication may also be dependent on accurate assessment of wound healing and wound tissue identification.

Wound assessments and measurements have long been fraught with subjectivity and considerable variability between clinicians [4]. Although there has been progress made in automated wound area measurements using computer vision and machine learning, the reporting of wound tissue composition and their reactive proportions is still largely subjective. When tissue compositions can be measured objectively, the results could improve the accuracy of wound healing progress monitoring, enable data-driven pressure injury staging, and better predict wound healing times. Therefore, the objectives of our work were, first, to measure the inter- and intrarater variability in manual tissue identification and quantification among a cohort of wound care clinicians. We sought to establish the extent of variability and subjectivity in manual wound tissue measurements and how this related to specific tissue types of interests. Second, we investigated if an objective assessment of tissue types (ie, size and amount) could be achieved using a machine learning model that predicts wound tissue types. The proposed model’s performance is reported in terms of numerical metrics, that is, mean intersection over union (mIOU) between model prediction and the ground truth labels. Finally, we evaluated the performance of the proposed model for wound tissue segmentation as collectively judged by a cohort of wound care clinicians observing the model predictions.

In this study, we proposed a fully automated wound and tissue segmentation technique based on deep convolutional neural networks. In *wound segmentation*, the goal is to delineate the region in the image that corresponds to the wound bed, and in *tissue segmentation*; the goal is to further breakdown regions within the identified wound bed into its constituent tissue types. The proposed deep learning models have been integrated into a mobile app and allow clinicians to obtain objective measurements, thereby eliminating the guesswork associated with wound tissue identification. This objective measurement addressed 2 challenges. First, differentiating tissue types within chronic wounds, when done manually, often varies between clinicians for a variety of reasons (eg, training and experience). Second, accurately determining the proportion or quantification (ie, measurement) of tissue types is challenging for a human. The models developed could automatically detect the location of a wound in an image, delineate the accurate boundaries of the wound, determine if any of the 4 types of tissue are present within the wound bed, and finally compute their relative proportions for reporting.

### Background and Related Work

For the context of this study, we aim to identify and quantify four major tissue types present in chronic wounds using deep learning: epithelial tissue, granulation tissue, slough, and eschar which are typically reported in wound assessment tools such as the Bates-Jensen Wound Assessment Tool (BWAT) [5] and to stage pressure ulcers using the National Pressure Injury Advisory Panel pressure injury staging system (Pressure Ulcer Scale for Healing [PUSH]) [6]. These tissues are present in an open wound in various color spectra when observed through a conventional imaging sensor. Epithelial tissue is observed as being pinkish or white regions that migrate from the wound margin with minimal exudate. It eventually covers the wound bed and is the final visual sign of healing. Granulation tissue is found mainly in the red spectrum. Its presence in a chronic wound indicates that regeneration is progressing well and that the wound is being properly treated. Slough is observed as a soft, yellow glutinous covering on the wound and is a type of necrotic tissue. Made up of dead cells and fibrin, a wound may be completely or partially filled with slough. It may also be fibrous or strand-like, adhering to the wound bed. Finally, because of tissue death, the surface of the wound is covered with a layer of dead or devitalized tissue (eschar) that is frequently black or brown. Initially soft, the dead tissue can lose moisture rapidly and become dehydrated with the surface becoming hard and dry. Colliquative necrosis are a subtype of this category and are yellow in color, similar to fibrin deposits. They are produced when the necrotic tissue softens and are, therefore, of a mushy consistency. The appearance of necrosis indicates degenerative breakdown of wound tissue.

The tissue composition and their relative quantity within the wound bed are important parameters for estimating wound healing progress. For example, the PUSH score [6] was proposed for pressure injuries and consists of three parameters: length×width, exudate amount (none, light, moderate, and heavy), and tissue type (necrotic tissue, slough, granulation tissue, epithelial tissue, and closed). Each parameter was scored, and the sum of the 3 scores yielded a total wound status score, which helped classify wound severity and identify nonhealing wounds. The relative quantities of relevant wound bed tissues are subjectively determined during assessments. As human beings, we are poor in accurately judging relative proportions, and inaccurate assessments can lead to incorrect downstream tasks like wound staging and treatment.

### Wound Area Measurement

There are numerous approaches [7] for wound area measurement, which vary in their accuracy and repeatability across multiple raters [8]. The most common approach would be to use a ruler and measure the width and length of a wound. This measurement does not allow accurate area measurement as wounds are not typically rectilinear in shape. The next step up could be to lay a grid pattern over the wound and mark the number of square grid boxes which overlap with the wound and thereby estimate the area.

Computer-aided approaches for wound segmentation (defining wound area) have been proposed in the past for small, controlled data sets, and their robustness on large-scale data sets has never been proven to be effective. Techniques include active contours [9], graph cuts [10], and color histograms [11] as well as machine learning approaches such as support vector machines [12,13] and artificial neural networks [14].

With the recent advances in artificial intelligence, it has now become possible to train a deep learning model to perform automatic segmentation of chronic wounds [15-17] in an end-to-end manner. These methods forego the need for image feature engineering and can automatically *learn* a hierarchy of image features required for a specific task. Despite the increasing number of papers being published for deep learning–based wound segmentation, most approaches have only been trained and tested on limited data sets often conducted in controlled settings as shown in Table 1. In addition, most of these approaches have not been demonstrated to run on mobile devices having limited computing resources—a critical factor in enabling objective electronic wound documentation at the bedside.

**Table 1:** Comparison of wound image data sets used for wound segmentation model training.

Study	Database used	Type	Total images in training set	Acquisition settings
Lu et al [16]	Medetec	Public	<500	Unspecified
Yadav et al [18]	Medetec	Public	77	Unspecified
Goyal et al [17]	Lancashire DFU^a^ database	Proprietary	600	Controlled, DSLR^b^, and flash used
Li et al [19]	Hospital+internet search	Proprietary	950	Unspecified
Chakraborty [20]	Medetec+proprietary data	Mixed	153	Unspecified
Wang et al [21]	NYU^c^ wound image database	Proprietary	500	Unspecified
Scebba et al [22]	SWISSWOU, Medtec, FUSC SIH^d^	Public	<300	Unspecified
This study	Swift Wound Data Set	Proprietary	Approximately 465,000	Uncontrolled and mobile phone camera

^a^DFU: diabetic foot ulcer.

^b^DSLR: digital single-lens reflex camera.

^c^NYU: New York University.

^d^SIH: secondary intention healing.

### Wound Tissue Segmentation

*Wound tissue segmentation*, which is a more challenging problem, has received far less attention than wound segmentation. Tissue segmentation entails a pixel-wise classification of various tissues that are found within the wound bed region. Past approaches have attempted to solve this using image patch–based color clustering or segmentation [18,20,23]. When classifiers are used, a training data set of image patches is built from a small set of wound images. The data set of image patches are then used to train a classifier to assign each patch to a specific tissue type. During inference, a wound image is first segmented from its background, and the wound region is split into smaller image patches. Each image patch is then classified by the trained model. This process is slow and typically not robust enough to handle variations in imaging conditions (eg, lighting and image angle) as is often the case in practice. Recently, deep learning techniques such as fully convolutional neural networks have been applied to wound tissue segmentation [19]. The approach is not fully automated; wound segmentation is performed using a dynamic color thresholding in the *YbCbCr* color space to segment the wound area, and then a fully convolutional neural network [24] is used to classify wound tissue within the segmented wound region. A limited set of images were used to train the network, and mobile implementation was not reported.

## Methods

### Overview

The inter- and intrarater agreement for wound tissue identification by wound care clinicians was first measured to establish the degree of variability present in visual estimation of tissue proportions and labeling tissue regions in wound images. The *Swift Wound Data Set*, which to the best of our knowledge is the largest labeled chronic wound data set for both wound segmentation and tissue segmentation ever reported in the literature for training deep neural networks for wound image segmentation and tissue segmentation, is described. Subsequently, a fully automated wound and tissue segmentation approach is presented, which is based on a deep encoder-decoder convolutional neural network and trained using data from our internal *Swift Wound Data Set*. Finally, the authors discuss the results obtained for both the interrater agreement study and the proposed deep learning technique for wound tissue segmentation in depth. A diagram depicting steps in this study is presented in Figure S1A-1 in Multimedia Appendix 1.

### Rater Agreement in Wound Tissue Identification and Quantification

To establish the variability of wound assessment when it comes to tissue region labeling, we examined the inter- and intrarater variability between wound care clinicians when estimating not only the presence of a given tissue type in the wound bed but also their relative proportions and their confidence in the estimations. In this paper, we interchangeably use the term *raters* to refer to the wound clinicians and nurses who were involved in our study.

**Figure 1:**
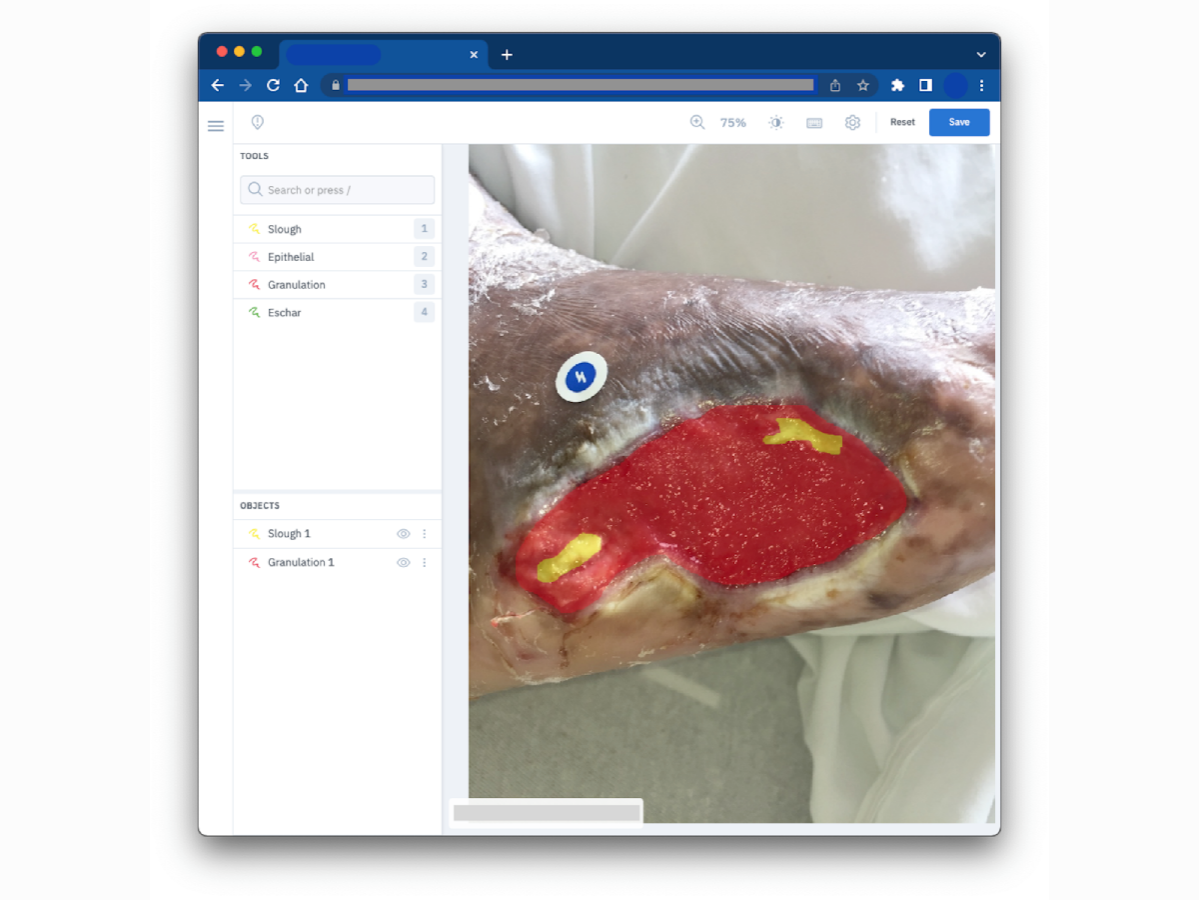
The web-based image annotation tool used for the interrater agreement study.

For this study, a random sample of 58 anonymized wound images (taken under uncontrolled lighting and viewing angles) from the *Swift Wound Data Set* consisting of pressure injuries, arterial ulcers, and venous ulcers was used. The data set was stratified according to skin tone using the Fitzpatrick scale and split into 3 subsets, with 50% overlap between subsets to measure intrarater agreement. In particular, 4 different tissue regions (epithelial, granulation, slough, and eschar) were manually labeled within the wound bed in each image. In addition, 5 experienced clinicians (a family physician, a dermatologist, a vascular surgeon, a burn surgeon, and a registered nurse) were tasked to label these images in random order using a browser-based image annotation tool shown in Figure 1. Apart from labeling (or annotating) the tissue regions, labelers were instructed to visually estimate the proportions of the 4 tissue types present within the wound bed and indicate their confidence levels when identifying these tissues.

To measure the inter- and intrarater variability, we used the Shrout and Fleiss CC [25]. As the same set of *k* raters labeled the same set of *n* samples in the data set, we used a 2-way, mixed effects model, specifically the intraclass correlation (ICC) as described in that paper to compute the ICC as a measure of reliability. Another measure of reliability is the Krippendorff α, which measures disagreement between a number of raters. For the sake of brevity, we refer readers to the study by Krippendorff [26] for a complete description of this statistical measure.

### Swift Medical Wound Data Set

Previous studies on wound segmentation were either trained or validated on small wound image data sets [18-23], often acquired under very controlled conditions and focused on a limited number of wound types; for example, diabetic foot ulcers were analyzed with high-resolution digital single-lens reflex cameras with large imaging sensors (23×15.6 mm^2^) and macrolenses; [17] however, questions remain as to the robustness of these approaches in real-world scenarios as results were demonstrated using very limited data.

In this study, we used our internal deidentified data set to train and validate the deep learning models for wound segmentation and tissue segmentation. Wound images in our data set were acquired using heterogeneous cellphone cameras under uncontrolled settings using the Swift Skin and Wound app from hundreds of skilled nursing facilities and long-term care centers across North America. Our data set is significantly larger (by 2 to 3 orders of magnitude) than data sets reported in previous studies [15,17,21,22] (Table 1). In addition, it is to be noted that there are no publicly available data sets for fully labeled wound tissues (ie, for epithelial, granulation, eschar, and slough), unlike the data sets listed in Table 1 which are data sets with purely binary labels (wound or background), which are much simpler to manually label.

**Figure 2:**
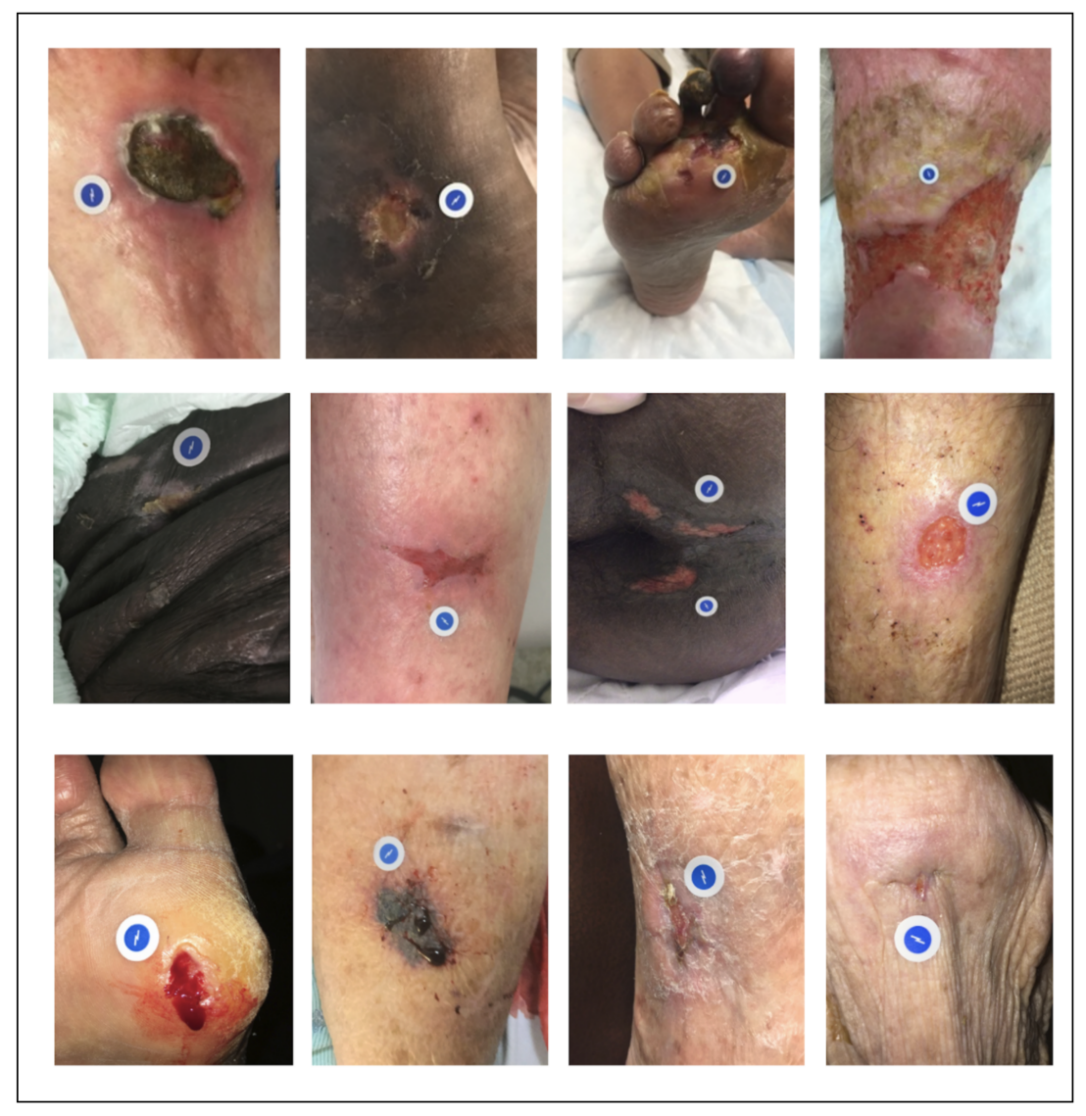
Sample images from the data set. The blue-white sticker seen in the images is the Food and Drug Administration–registered HealX calibrant used with the Swift Skin and Wound app for color-correction and scale calibration. Note variations in terms of viewing angles and distances, background, wound types, severity, skin tone, and wound sizes.

There is significant variability in terms of viewing angles, lighting conditions, background, and magnification factors for wound images in the Swift data set as shown in Figure 2. This data set also covers a wider range of skin lesions and chronic wounds than any of the previously published studies. Specifically, it consists of 14 different types of wounds or skin lesions at various stages of healing. These include bruise or abrasion, blister, burn, cancer lesion, diabetic foot ulcer, laceration, moisture associated skin damage, mole, open lesion, pressure injury, venous ulcer, rash, skin tear, and surgical wound.

There are numerous variations in skin tones because of ethnicity, which makes it challenging to isolate healthy skin and wound bed regions using traditional computer vision techniques. In most images in the data set, healthy skin area was found to be covered with age spots—a pigmentation effect associated with older individuals. Figure 2 shows examples of skin tone variations present in the data set. There is also a wide variation of the visible characteristics of the wounds in our data set; for example in terms of wound type, location, and severity.

This data set reflects the actual diversity in wound images typically observed in practice. As deep learning techniques generally scale well and perform better when trained with larger data sets, the automatic wound and tissue segmentation approach presented in this paper are expected to be more robust and accurate than previously reported approaches for large-scale deployment. It is to be noted that owing to patient privacy concerns, we are unable to publicly share the data sets used to train our models.

### Fully Automated Wound Tissue Segmentation

**Figure 3:**
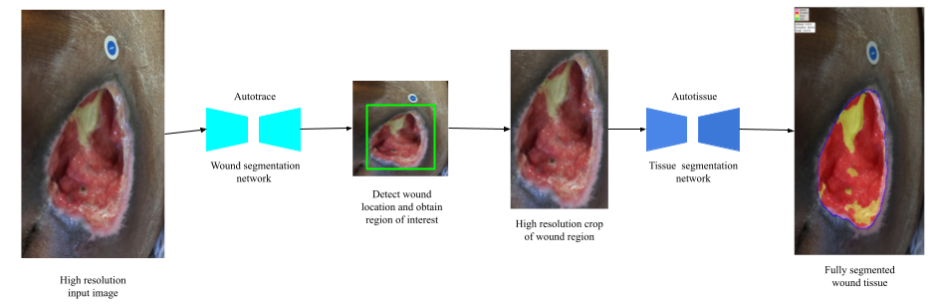
A high-level overview of AutoTissue, the proposed fully-automated wound and tissue segmentation approach.

A high-level depiction of this fully automated wound and tissue segmentation method is shown in Figure 3. A high-resolution image is first acquired using the smartphone camera. The first stage involves detecting the presence of a wound and determining the bounding box of the wound. Although other reported approaches [21] used a separate object detection model such as YoloV3 [27] to locate the wound, we used an encoder-decoder wound segmentation network, dubbed *AutoTrace*, whose predictions can not only be used to compute the bounding box of the wound, but also determine the accurate segmentation (trace) of the wound bed. This model is small and fast enough to enable real-time inference on mobile devices.

**Figure 4:**
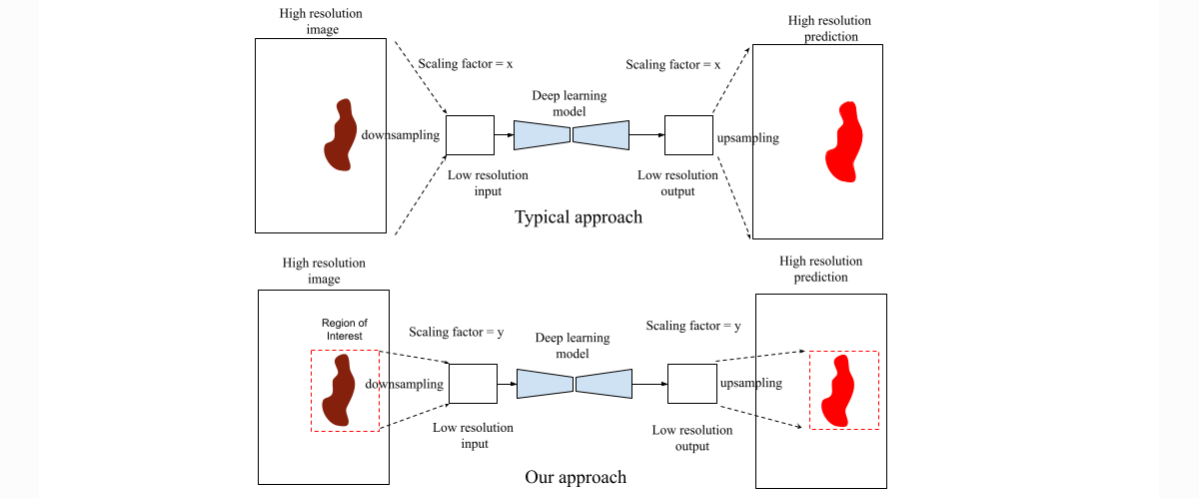
Diagram depicting the relationship between scaling factor and potential information loss owing to downscaling and the proposed approach. Note that scaling factor x is much larger than y as shown in the diagram.

When implementing deep learning architectures, particularly on mobile devices, the input image dimension is a critical factor to ensure that memory and computation requirements are manageable. Therefore, deep learning approaches typically use a low-resolution version of the images for training and inference. A drawback of using low-resolution image inputs is that information loss is attributed to downscaling. The higher the downscaling factor, the higher the possibility of information loss owing to interpolation (see Figure 4 for an illustration). To ensure that we minimize the potential information loss from image scaling or subsampling, we take steps to apply our deep learning models on regions of interest in an image, particularly at locations where an actual wound is located within the image. Therefore, we first use the *AutoTrace* model to detect the presence of an open wound in the image, then select a bounding box encompassing the detected wound region, and finally rescale that region to the dimension required as inputs to our tissue segmentation model, that is, *AutoTissue.*

Because a wound typically only constitutes between 25% and 65% of the imaged area in our data set, applying our tissue segmentation model directly on the detected wound region ensures a high wound-to-background pixel ratio for the model inputs, which leads to more accurate predictions and less errors from the series of downsampling and upsampling operations which are applied when using the deep learning models.

The tissue segmentation network, *AutoTissue,* produces a dense prediction of 4 wound tissue types (epithelial, granulation, slough, and eschar) when present within the detected wound bed. Wound border refinement is made using the wound contour computed from *AutoTrace’s* wound prediction. Here, we clip the predicted tissue predictions with the accurate wound contour to ensure only tissues that are present within the wound bed are used to compute the tissue proportions.

In the following subsections, we present a high-level overview of both the *AutoTrace* and *AutoTissue* models.

### AutoTrace: Wound Segmentation Model

**Figure 5:**
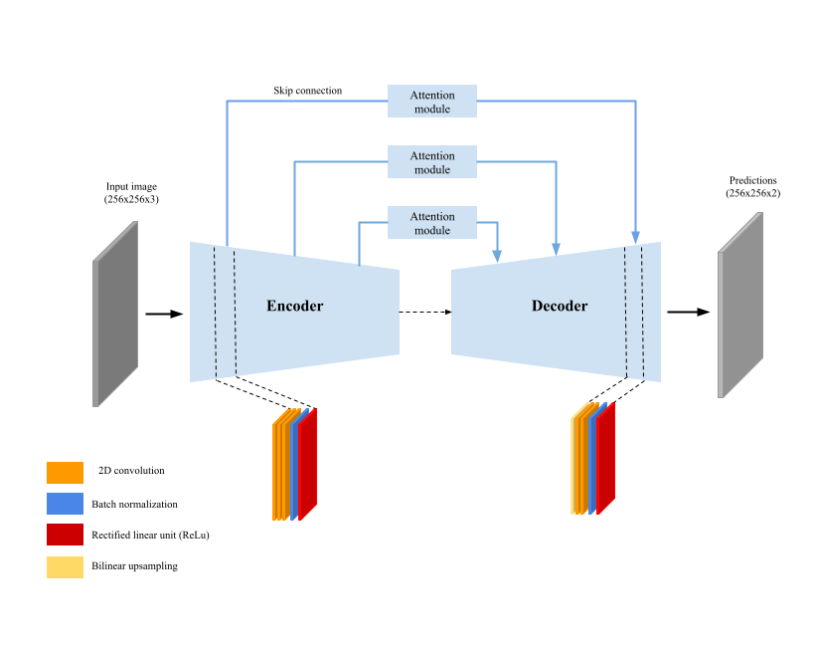
A graphical representation of the AutoTrace model for wound segmentation. ReLu: Rectified Linear Unit.

Our wound segmentation model, as depicted in Figure 5, is a deep convolutional encoder-decoder neural network with attention gates in the skip connections. The encoder block is responsible for feature extraction, and the decoder block *decodes* the learned features to produce the required output (ie, the segmentation mask). The *AutoTrace* architecture was derived from the study by Schlemper et al [28] who first proposed attention gates in convolutional neural networks. Additional customizations were implemented in our models to allow them to run on mobile devices. We replaced the normal convolutional blocks with depth-wise separable convolutional layers [29]. The main advantage of replacing normal convolutions with depth-wise separable convolutions is the significant reduction in computation required with only a small penalty to the final accuracy. Second, we implemented strided depth-wise convolutions that can *learn* to downsample activations instead of a fixed max-pooling operation for downsampling. Third, an additive attention gate was placed in each of the skip connections in the architecture. The inclusion of additive self-attention modules in the skip connections regulates the flow of activations from earlier layers. The attention coefficients identify salient image regions and prune feature responses to preserve only the activations relevant to the specific task. This ultimately provides improved performance for the wound segmentation task. Finally, to further reduce computational and memory requirements, the decoder blocks consisted of a bilinear upsampling followed by 2 depth-wise separable convolution layers per block instead of transposed convolution layers.

We trained this model on 467,000 image-label pairs with wound region labels provided by clinicians. Our held-out test set consists of 2000 image-label pairs of arterial, venous, pressure, and diabetic ulcers taken in diverse imaging conditions and wound locations. During training, data augmentation performed included random crops, horizontal or vertical flips and random contrast and brightness adjustments. Unlike the U-Net with Attention model [28] which was trained using deep supervision, we trained our model using a single loss function by minimizing the soft dice loss which is the form of the following:



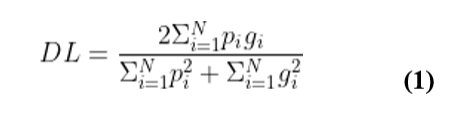



where 
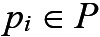
 is the predicted probability of the pixel and 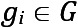 is the ground truth of the pixel. The early stopping criterion was used to stop the training after convergence. L2 regularization and dropout regularization [30] were used to control overfitting.

### AutoTissue: Tissue Segmentation Model

**Figure 6:**
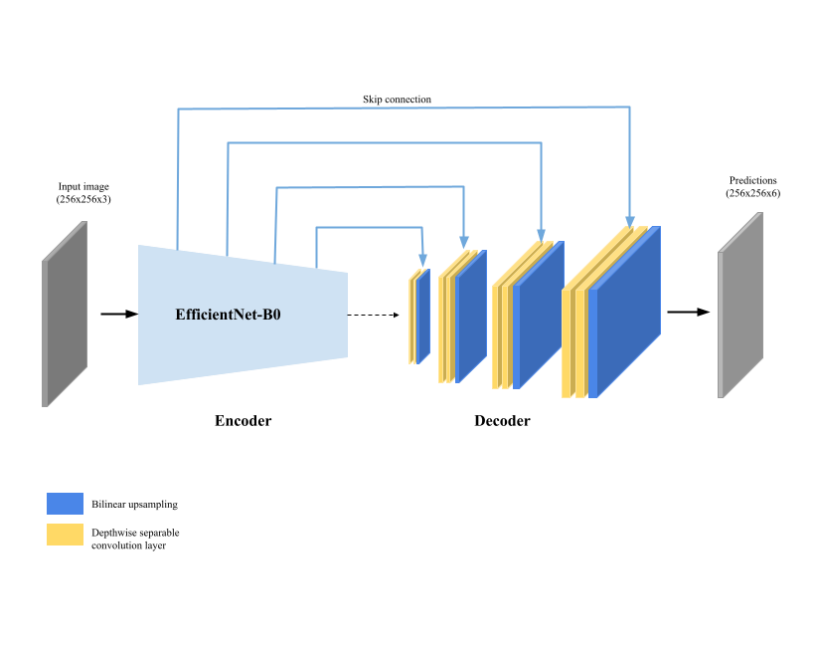
Graphical representation of the AutoTissue architecture for wound tissue segmentation.

The wound tissue segmentation model presented in this paper is significant as most of the previously published studies using deep learning in the domain focused on wound segmentation [15-18,20] and not tissue segmentation. The *AutoTissue* model (shown in Figure 6) is an encoder-decoder convolutional neural network that uses an *EfficientNetB0* architecture [31] as the encoder. The decoder is made up of 4 blocks; each of which consists of a single 2-dimensional bilinear upsampling layer followed by 2 depth-wise convolution layers.

The *AutoTissue* model was trained using a subset of 17,000 anonymized wound images from the *Swift Wound Data Set* where healthy tissue, background, the HealX calibrant sticker, and the 4 wound bed tissue regions, if present, were labeled in the images. The data set was meticulously labeled by a team of trained labelers and was curated by a panel of wound clinicians. The authors could not identify any published work that used labeled data at this scale for deep learning–based wound tissue segmentation in the literature. Data augmentation, a technique used to increase the amount of training data and prevent overfitting when training deep learning models, was performed on the fly, during model training by applying random crop and rotation, random color jittering and cutout regularization [32]. Both networks were trained using AdamW (Adam With Decoupled Weight Decay) [33] adaptive learning rate using an initial learning rate of 0.001. The held-out test set consisted of 383 images consisting of stage-2 pressure, arterial, and venous ulcers and diabetic wounds.

## Results

### Interrater Agreement Study Results

First, the authors presented the results obtained from the interrater agreement study. As mentioned earlier, the data set was split into 3 subsets, and each subset was presented to the raters (clinicians) for labeling at 1-week intervals. In all, 50% (29/58) of the images were labeled thrice, each presented 1 week apart to measure intrarater agreement. From each manually labeled image, the tissue proportions within the wound bed were assessed by counting the pixels that belonged to a certain class and computing its proportion against the total wound area. In addition, visual estimation of the tissue proportions was also recorded.

**Figure 7:**
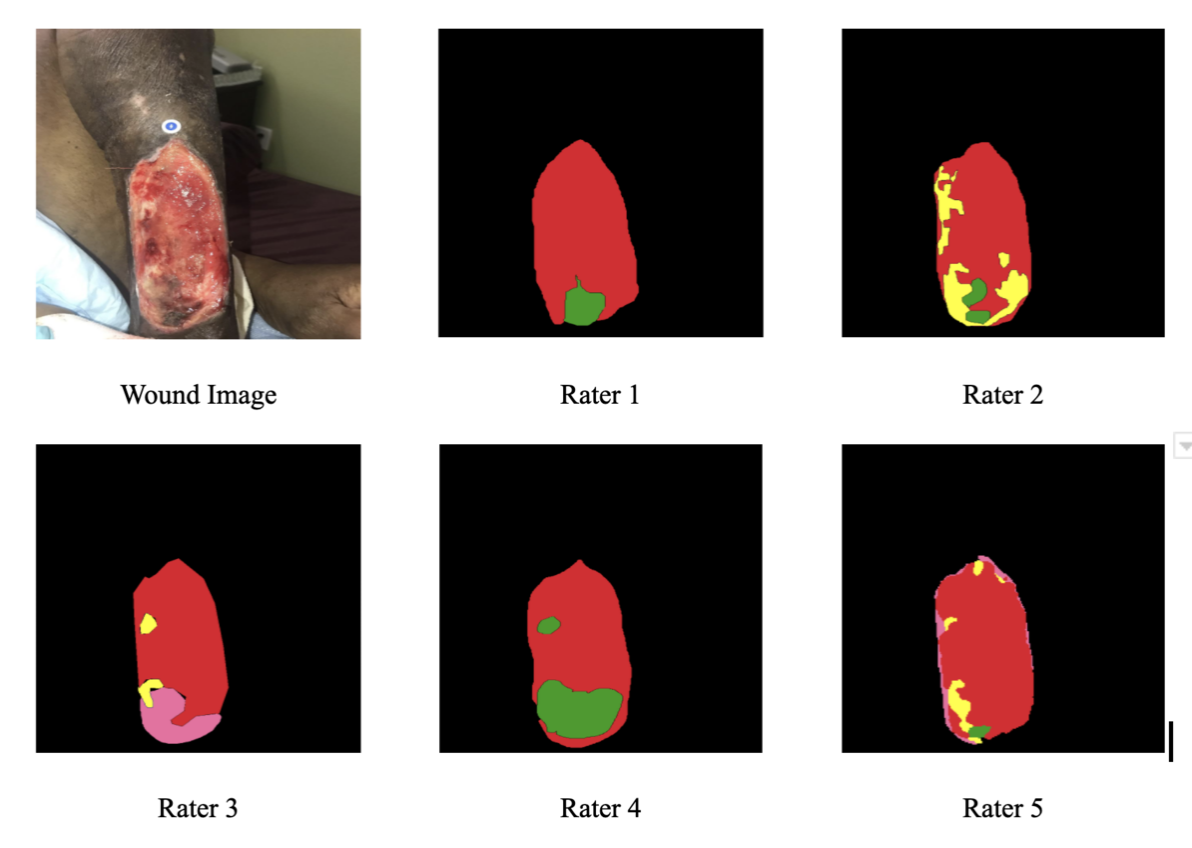
The variability between raters in labeling different tissue regions is visualized in this figure. The colors of the labels correspond to different tissue types: red corresponding to granulation, pink to epithelial, yellow to slough, and green to eschar.

Figure 7 illustrates an example set of labels made by the wound clinicians in our study. Note the considerable variability between the labels and this observation in this example. A similar observation extends to the entire set of 58 images used to study the interrater variability and is captured by the interrater agreement ICC score presented in Table 2 Additional examples of inter- and intrarater variability in labeling tissue are provided in Multimedia Appendix 1.

The intrarater agreement for computed tissue proportions is presented in Table 3. As mentioned earlier in this section, a subset of wound images were repeatedly presented to the clinicians to label at 1-week intervals. Thus, each image has a set of 3 labels provided by the same clinician.

**Table 2:** Interrater agreement intraclass correlation for tissue proportions that were computed from wound images labeled by wound clinicians in our study.

Tissue type	Intraclass correlation
Epithelial	0.389
Granulation	0.765
Slough	0.591
Eschar	0.759

**Table 3:** Intrarater intraclass correlation for tissue proportions that were computed from wound images labeled by wound clinicians in our study.

Rater	Epithelial	Granulation	Slough	Eschar
Rater 1	0.785	0.789	0.843	0.803
Rater 2	0.410	0.685	0.836	0.840
Rater 3	0.535	0.729	0.641	0.493
Rater 4	0.475	0.806	0.745	0.809
Rater 5	0.757	0.958	0.986	0.963

As can be seen in Table 3, the high ICC score for individual raters (rows in the table) signifies that raters were relatively consistent when labeling (thereby reflected by computed tissue proportions) the same image multiple times over a period. The only exception was the relatively poorer intrarater agreement for epithelial tissue labeling compared with other tissue types.

Although there was moderate to high agreement in the *intrarater* agreement, only moderate *interrater* agreement was observed between raters when labeling tissue types in wound images. In particular, interrater agreement was poor for epithelial and slough tissues and moderate for other tissues based on ICC, values shown in Table 2. Note that the computation of tissue proportions as identified by experts as performed in this study differs from how tissue proportions are *visually estimated* in practice, which can be extremely subjective. The images were labeled using a browser-based image annotation tool that allows precise annotation of different tissue types; however, in practice, wound clinicians do not have the time or tools to perform the same. We can, therefore, anticipate even higher inter- and intrarater variability in subjective visual estimations of tissue proportions compared with that reported in this study.

**Figure 8:**
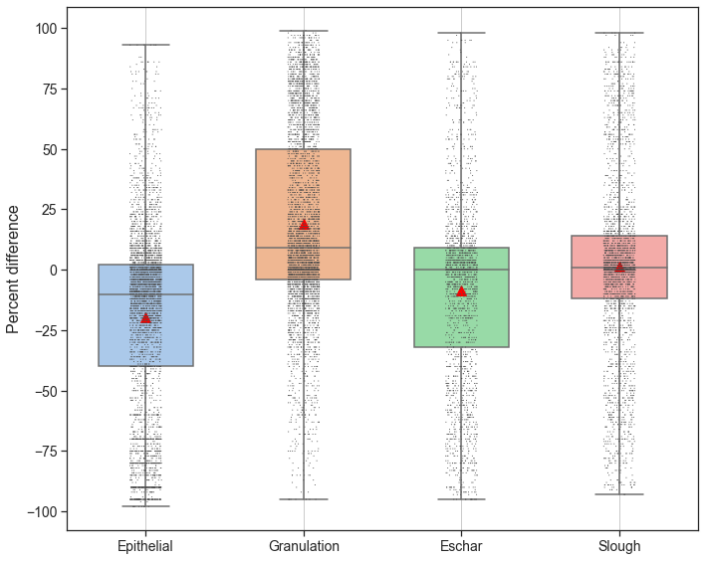
Box plot showing difference (in percentages) between computed tissue proportions and visual estimates for different tissue types in the rater agreement study. Negative differences indicate overestimation of rater’s visual estimation. Scatter plot shows actual distribution of data points for computed differences in tissue proportions. Red triangle point indicates the mean.

Figure 8 shows a box plot depicting the differences between the computed proportions based on labeled regions and the visual estimates of 4 different tissues present in the set of 58 labeled wound images as labeled in the inter- and intrarater agreement study. The subjectivity in visual estimation naturally leads to variability between the rater’s visual estimates and the proportions computed from tissue labels provided by the raters through the image annotation tool. Raters largely overestimated epithelization and eschar (shown by mean and median of the box plot being negative values) and underestimated granulation and slough during visual estimation. There is substantial variability (in terms of SDs) in the distribution of errors between estimation and computed proportions for all tissue types in the range of 38% to 39%.

Apart from measuring inter- and intrarater agreement for tissue proportions calculated from labeled tissue regions, we additionally computed the interrater agreement in the clinician’s ability to identify the presence of any 1 of the 4 tissue types in the wound images presented to them. As this involves binary decisions (ie, *True* when a given tissue is labeled as present and *False* when it is not—disregarding the proportions computed), we used the Krippendorff α to measure the interrater agreement. Our results, as presented in Table 4, indicate very poor interrater agreement in determining the presence and regions of epithelial tissue with a Krippendorff α value of only .014, whereas fair to moderate agreement was scored for the other tissue types. Granulation was the most agreed upon tissue type, which was in line with observations in clinical practice.

**Table 4:** Interrater agreement in identifying wound tissue types.

Measure of reliability	Epithelial	Granulation	Slough	Eschar
Krippendorff α	.014	.664	.415	.379

**Figure 9:**
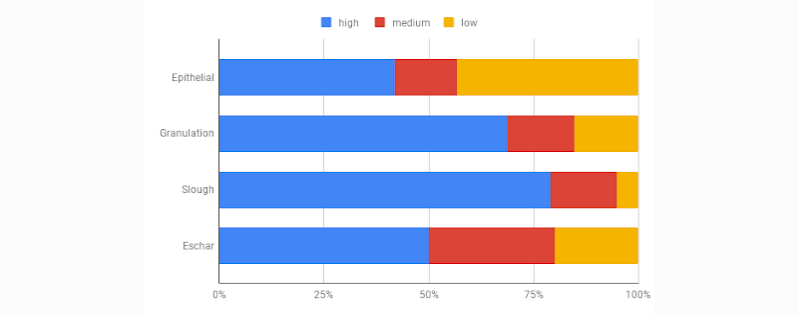
Clinician’s confidence in tissue identification.

One final parameter that we captured during this study was each clinician’s confidence in labeling the 4 tissue types in question. Results indicate that the clinicians involved in the study were generally very confident in labeling granulation and slough tissues, moderately confident when labeling eschar, and least confident when labeling epithelial tissue as shown in Figure 9. This result correlated well with the Krippendorff α value we observed in Table 4. The significance of this observation will be seen later when we discuss our model performance for different tissue types. Data and code pertaining to these experiments is available for public download on the web [34].

### Automated Wound and Tissue Segmentation Results

**Figure 10:**
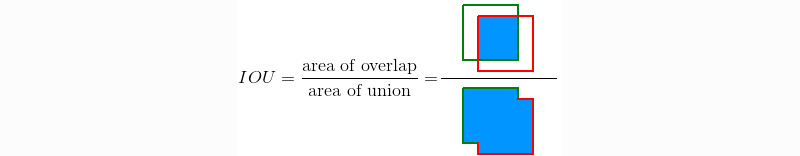
A graphical representation of mean intersection over union (IOU) which varies from 0.0 (no overlap) to 1.0 (perfect overlap).

The performances of both the wound segmentation and wound tissue segmentation models were evaluated separately on 2 different held-out test sets. We evaluated the performances of our models by computing an objective numerical metric, which is the mIOU between the ground truth labels and the predictions made by our models. The intersection over union metric measures the number of pixels common between the target and prediction label masks divided by the total number of pixels present across both label masks (see Figure 10 for a graphical depiction). When there are several classes of labels involved (eg, in the case of wound segmentation, there are two classes to be predicted, ie, wound and background classes), then the mean value of individual per class intersection over union is computed to arrive at a single metric, which is the mIOU.

**Figure 11:**
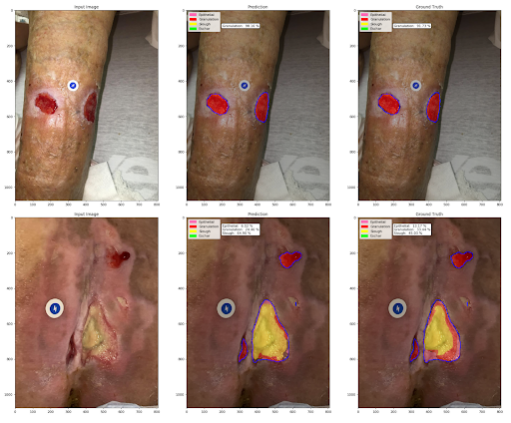
Sample wound and tissue segmentation results. (left to right: input image, model prediction, and ground truth). The blue contour is the wound region as determined by the AutoTrace model.

The wound segmentation model, *AutoTrace*, achieves a mIOU of *0.8644* for wound region segmentation, whereas the *AutoTissue* model achieves a mIOU of *0.7192* for tissue segmentation on the held-out test sets. Several sample predictions made using our technique are presented in Figure 11. See Figure S1C in Multimedia Appendix 1 for additional results

**Figure 12:**
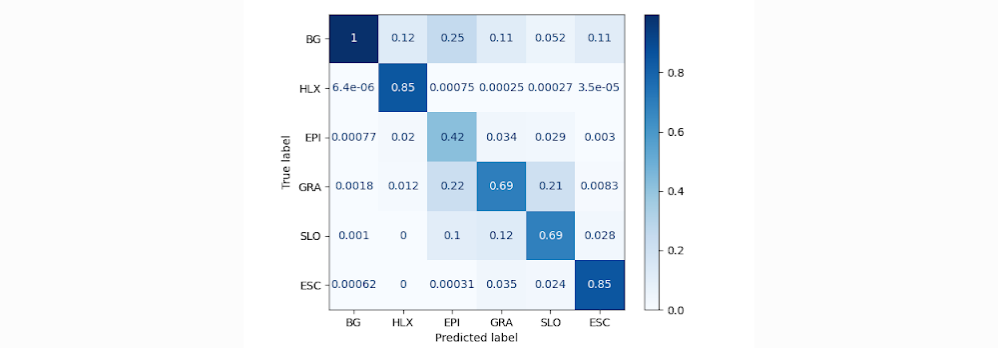
Normalized confusion matrix for the AutoTissue model on the held-out test set. Background (BG) includes all nonwound bed pixels including healthy tissue and background, HLX represents the calibrant sticker used for computing accurate wound measurement. EPI: epithelial; ESC: eschar; GRA: granulation; SLO: slough.

**Figure 13:**
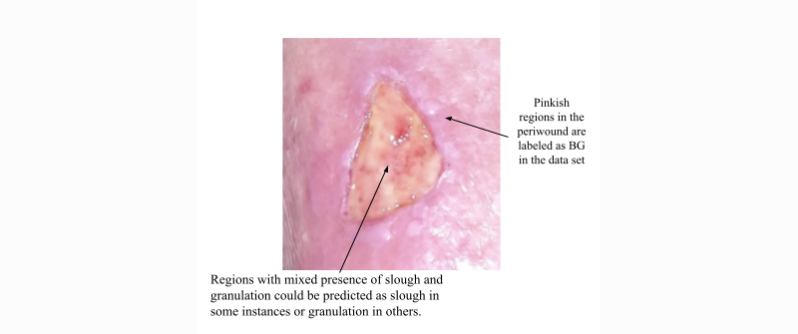
A wound region where pinkish tissue outside the wound bed is labeled as background in our training data set, as we are only interested in tissues within the wound bed. Note that these pinkish regions share similar appearance as tissues belonging to the epithelial class. BG: background.

The normalized confusion matrix for the model predictions is shown in Figure 12. The confusion matrix indicates that the *AutoTissue* model is able to accurately distinguish between the wound region and healthy skin or background. Similarly, the model is performant when segmenting the HealX calibrant sticker as its appearance is relatively consistent on all images. Granulation, slough, and eschar tissue prediction performance is also favorable. We can observe that slough is largely misclassified as granulation and vice versa. This primarily reflects the challenges faced by wound clinicians when labeling regions in the wound bed that show the mixed presence of slough and granulation tissue where labelers tend to be less confident or inconsistent across different images as shown in Figure 13.

Table 5 presents the classifier report for the *AutoTissue* model. As noted, the epithelial classification exhibits low precision and low recall and a corresponding *F*_1_-score of only 0.253. In contrast, the *F*_1_-score for slough and eschar detection is relatively high, registering values of 0.731 and 0.802, respectively.

On the test set, the model correctly predicts 42% of pixels belonging to epithelial tissue; however, at the same time confuses epithelial tissue with healthy skin, which is part of the background or nonwound class. We attribute the model’s poor performance for this class of wound tissue to the fact that the epithelial class is underrepresented in the training data set and it is challenging to label correctly within the wound bed region. Because tissue proportions are computed for regions within an open wound, epithelial tissue found in the periwound region is not considered when computing the proportions. In addition, we observed in our study that there is very poor agreement between raters in labeling epithelialization within the wound bed.

We can note that slough is sometimes misclassified as granulation and vice versa. This primarily reflects the challenges faced by wound clinicians when labeling regions in the wound bed that show the mixed presence of slough and granulation tissue where there could be considerable disagreement between raters.

Apart from testing model performance on the held-out test data as is typically reported in machine learning literature, we additionally used the 58 images which were part of the interrater agreement study to measure the agreement between predictions of the AutoTissue model and wound clinicians’ labels in terms of the mIOU metric. From Table 6, we note that there is a relatively high degree of agreement for the intersection over union metric for all tissue types between our model’s output and the clinicians’ ground truth segmentation. In other words, there is high consistency between our model’s segmentation results compared with experts’ labels.

Finally, we measured a consensus-based evaluation of the correctness of the predictions made by our deep learning models. The set of 58 images and corresponding *AutoTissue* wound tissue predictions were shown to the group of wound clinicians. We then requested the wound clinicians to collectively examine the model’s predictions, discuss, and provide a quality rating based on a consensus-based agreement. This approach demonstrated that 91% (53/58) of the images were jointly rated as being very good to fair, and only 9% (5/58) of the predictions were rated as being poor. This provides an additional validation of the plausibility of our model predictions on the 4 tissue types present within the wound bed.

**Table 5:** Classification report for the AutoTissue model.

Metric	Epithelial	Granulation	Slough	Eschar	Average
Precision	0.180	0.623	0.783	0.759	0.586
Recall	0.424	0.693	0.685	0.850	0.663
*F*_1_	0.253	0.656	0.731	0.802	0.610
Sensitivity	0.424	0.693	0.685	0.850	0.663
Specificity	0.603	0.772	0.862	0.825	0.765

**Table 6:** Interrater agreement intraclass correlation for per-tissue intersection over union between AutoTissue and expert labelers.

Tissue type	Intraclass correlation
Epithelial	0.764
Granulation	0.861
Slough	0.736
Eschar	0.855

## Discussion

### Principal Findings

Although we have established that there is a considerable variability in tissue labeling even between trained wound clinicians, it might appear contradictory that a deep learning model that has been trained using *noisy* labels can perform as well as humans. Arpit et al [35] suggested that sufficiently large deep neural networks did not memorize the data when trained on data sets that had mostly correct labels. Multiple studies [36-38] have also shown that a machine learning model trained using a large-scale data set of nonexpert labels can still match the performance of experts in medical image segmentation. During training, these models learn the dominant patterns observed in the data set that are shared across the data set. Therefore, to put this into the context of our own model, as the labels in our data set are pixel-wise labels (ie, there exists a label for each pixel in the image), there is an overwhelming majority of pixels that do have correct labels associated to them and a small percentage of pixels that have wrong labels associated to them owing to interrater variability. However, the *noise* that may be present in our labels is generally distributed across images and raters. Despite this, our models have the capacity to learn the dominant features for each of the tissue types and therefore are able to generalize well to unseen instances owing to the distributed and hierarchical representation, which is inherent in the design of deep neural network architectures and aided by the regularization schemes (eg, cutout and L2 regularizations) that we implement when training the models. Training on a very large data set, as was the case in our study, helped mitigate the effects of *noisy* or inaccurate pixel-wise labels. In future studies, we would want to pursue several methods [39] for dealing with noisy labels in training data to further improve our segmentation results. The labeling confidence we observed for different tissue types has direct implications to the performance of the models. We note that the model performs well for tissue classes that are easier to label and for which there are less ambiguities among labelers, and vice versa.

Still a major issue within the dermatology and medical community in general is that physicians are not trained to assess dark skin well, including wounds because most medical textbooks have illustrations that feature predominantly light-colored skin, and physicians still face a huge challenge in detecting certain tissues in dark skinned individuals. An unfortunate outcome of this implicit bias is that an accurate and prompt diagnosis may not always be possible with individuals of the Black, Indigenous, and people of color communities. For example, necrosis at the wound edge, which is an important finding, is still challenging to identify on dark skin. Our wound and tissue segmentation models on the other hand have been trained on a diverse set of wound types and skin tones, and this is a major step in ensuring the models do not inadvertently learn a bias toward a particular skin tone, which naturally leads to enhancing the ability to care for all patients.

Timely, accurate wound assessment and reporting is important for modern wound care practice. Rather than paper-based measurements and wound assessments, electronic wound assessments could have a large impact on the wound healing progress as such systems provide a more objective wound measurement, allow tracking of wound healing progress, and minimize errors or incomplete assessments. We believe that with the current technological advances, smartphone-based wound assessments will continue to have an increasing footprint in modern wound care practice. Therefore, we prioritized designing our deep learning models to be able to execute on a wide range of off-the-shelf smartphones, without the need for off-line processing. Both the *AutoTrace* and *AutoTissue* models have been integrated into the Swift Skin and Wound app and are very performant for real-time inference on mobile devices (Multimedia Appendix 2). Both models have a combined size of <16 million parameters and a peak memory consumption of approximately 85 MB per model during inference. The inference time averaged 300 ms on mobile central processing units when tested on low-end devices and is considerably faster on newer devices that support graphics processing unit acceleration.

### Limitations

The relatively poor performance of the model for epithelial tissue can be attributed to several factors, including the challenge in labeling epithelialization within the wound bed and the distinction between *epithelialization* and *epithelial tissue*, that is, intact skin resulting from healing process in the periwound region, which also appears pinkish but is not considered during labeling. While acknowledging this limitation, we believe that there would be no significant impact on pressure ulcer staging (PUSH) or wound assessment (BWAT) because epithelialization is not critical for these measures.

The Swift Skin and Wound app used for measuring and documenting wounds and the HealX fiducial markers are each Food and Drug Administration–registered Class I medical devices which are being used in over 4000 organizations across North America. We agree that verification, validation, and continued monitoring of artificial intelligence performance, including understanding the way outputs are used are critical and are the core to deployment of such models. This study documents some, but not all, of the extensive validation undertaken as part of our design, development, risk management, and regulatory processes. The deep learning models reported in this manuscript have been integrated into the Skin and Wound device app but are yet to be deployed on a wide scale in these organizations. The predictions from this model serve to assist clinicians to document wounds in an objective manner given that our inter- and intrarater studies have shown that there is a high degree of variability when humans manually detect and estimate wound tissue proportions. In the mobile app, the models’ outputs serve an informative role and do not constitute diagnosis or therapy. All Swift Skin and Wound users are provided both live training and training materials, wherein these issues are addressed. We continue to monitor the performance of all models after deployment and regularly assess all our products to determine whether changes should result in reclassification or premarket notification or authorization. The models discussed in this work were assessed through our regulatory process to not cause such a change in classification.

### Conclusions

Significant interrater variability in visual estimation of tissue proportions by a cohort of wound care clinicians reflects the subjective challenge of tissue typing. Epithelialization is the most varied measurement and can be linked to the challenges observed in clinical practice in identifying that tissue type. To reduce ambiguity and provide objectivity in tissue identification, we present a framework for deep learning–based wound segmentation and tissue segmentation that is capable of running in near real time on off-the-shelf smartphones. To the best of our knowledge, our models have been trained using a chronic wound image data set that is not only magnitudes larger than any previously reported data sets but also the most diverse in terms of wound types and skin tones allowing for unbiased, robust models to be trained. These models are able to provide plausible predictions of tissue types and allow accurate and objective tissue proportions to be computed. This will help to improve objectivity in downstream tasks such as pressure ulcer staging, healing risk prediction, identification on nonhealing wounds, adjustment of treatment options and may ultimately lead to improved healing rates for chronic wounds.

## Appendix

Multimedia Appendix 1 Overview of research approach and additional results.

Multimedia Appendix 2 Screen capture of our mobile app implementation of wound tissue segmentation.

## Abbreviations

BWAT: Bates-Jensen Wound Assessment Tool
ICC: intraclass correlation
mIOU: mean intersection over union
PUSH: Pressure Ulcer Scale for Healing
