# Supplementary material for: Fully Automated Wound Tissue Segmentation Using Deep Learning on Mobile Devices: Cohort Study
Source: JMIR Mhealth Uhealth. 2022 Apr 22;10(4):e36977. doi: 10.2196/36977 (PMC9077502; doi:10.2196/36977)

Appendix 1A

Figure S1A-1. Diagram summarizing all steps carried out in this research.

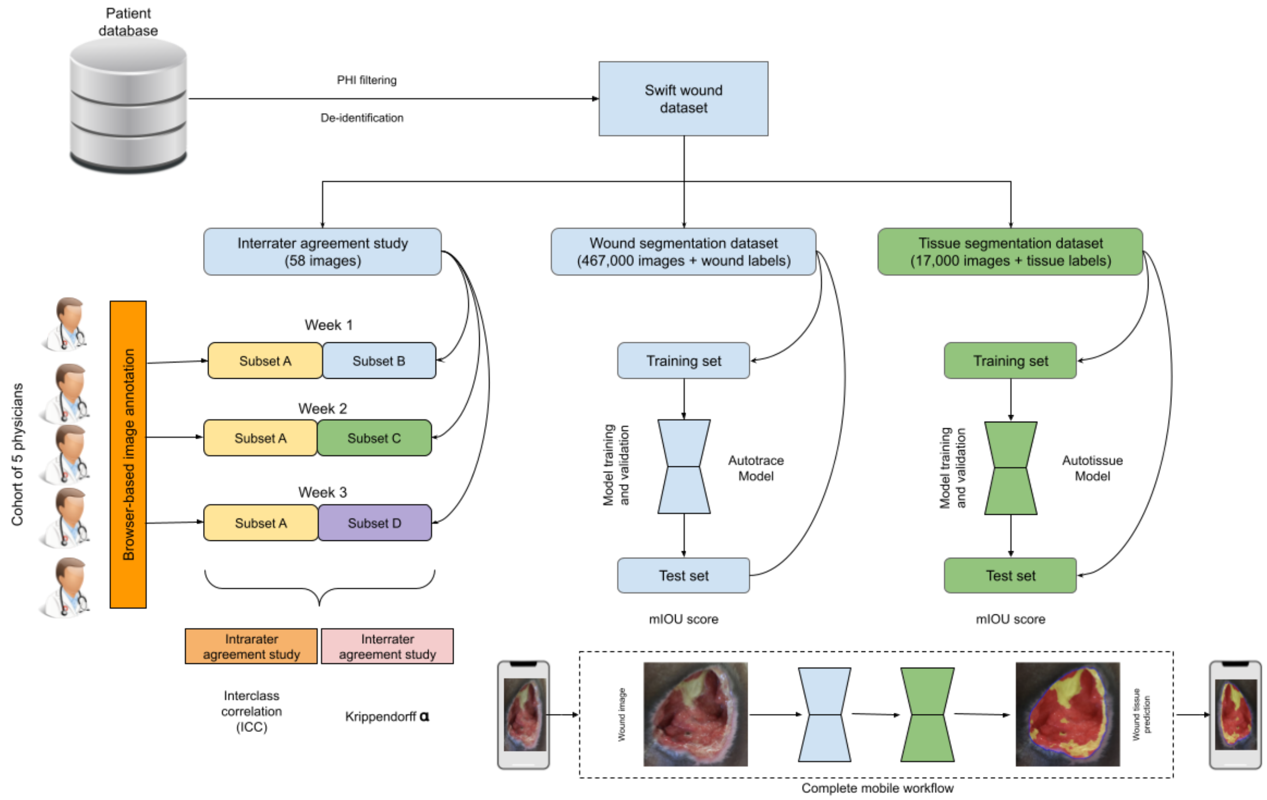

Figure S1A-2. Example of variability observed in tissue labeling between clinicians in our study. Pink - Epithelial, Red- Granulation, Yellow - Slough, Green - Eschar

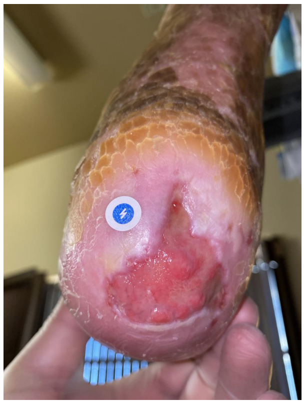	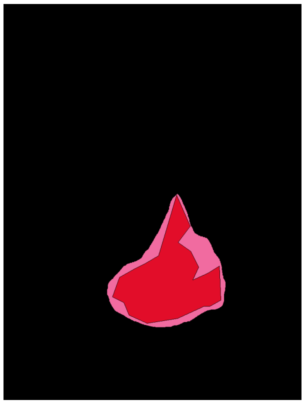	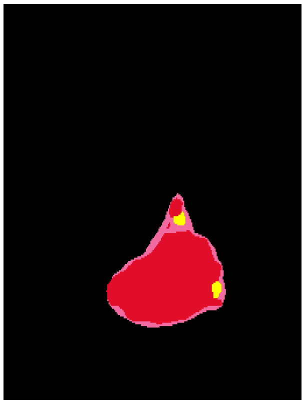
Wound Image	Rater 1	Rater 2
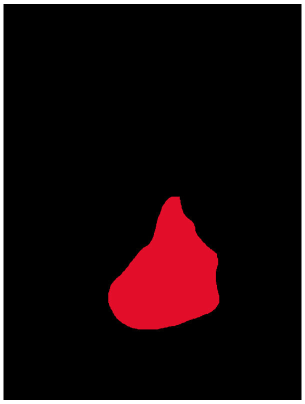	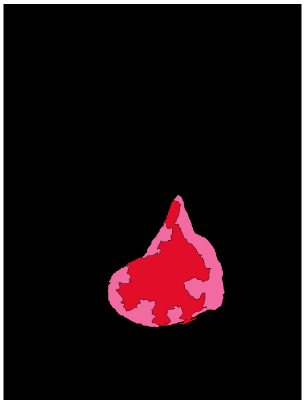	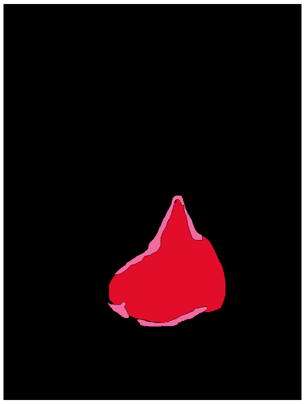
Rater 3	Rater 4	Rater 5

Figure S1A-3. Second example of variability observed in tissue labeling between clinicians in our study. Pink - Epithelial, Red- Granulation, Yellow - Slough, Green - Eschar

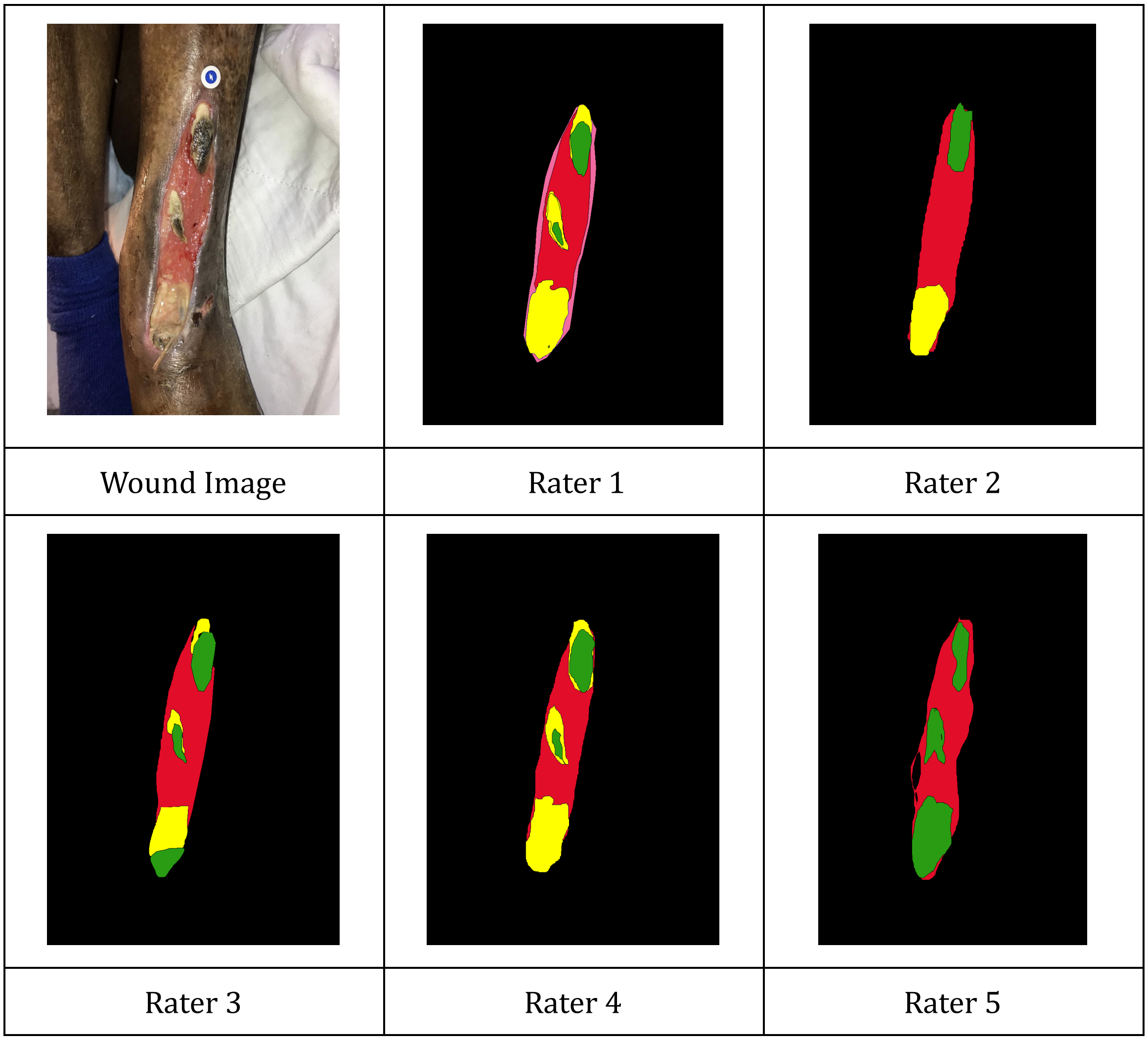

Figure S1A-4. Third example of variability observed in tissue labeling between clinicians in our study. Pink - Epithelial, Red- Granulation, Yellow - Slough, Green - Eschar

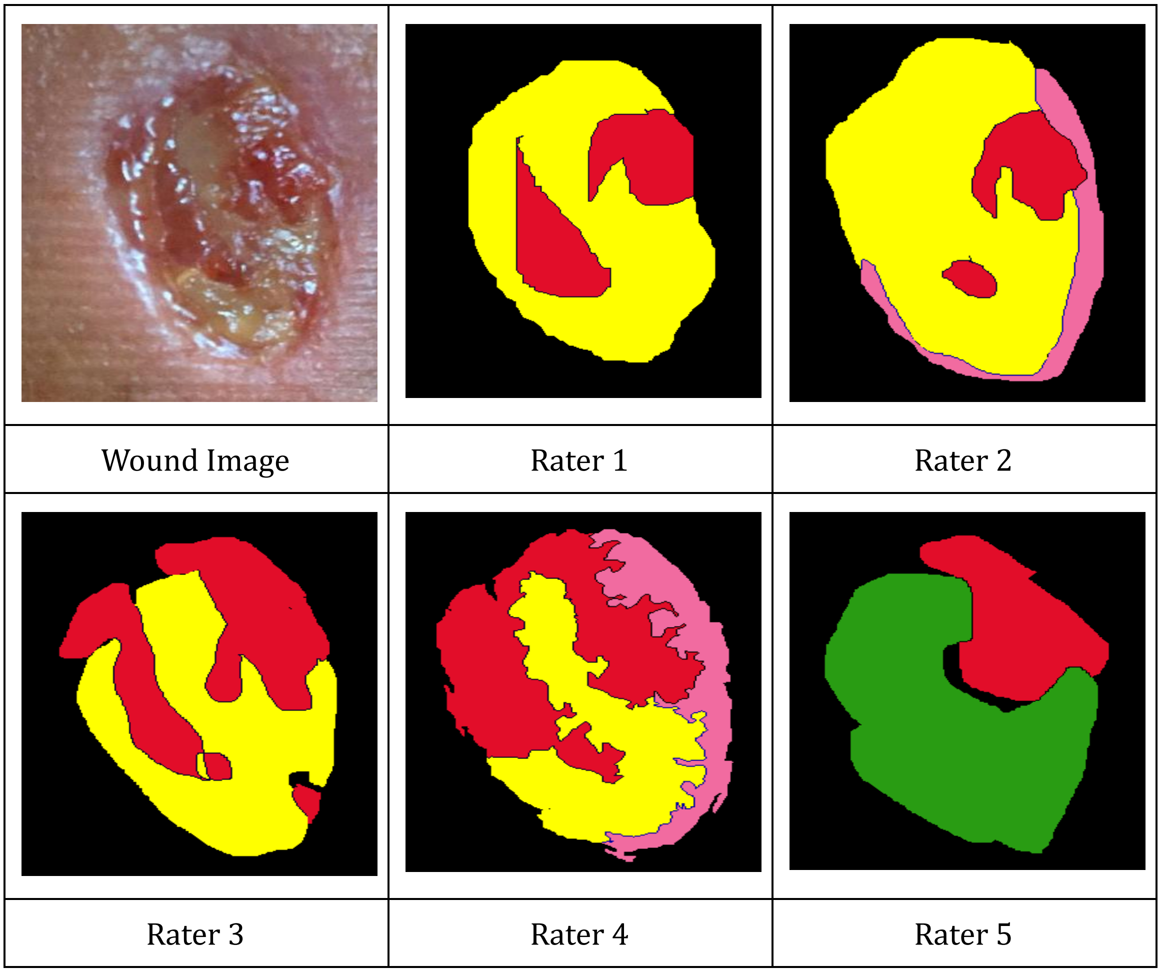

Appendix 1B

Figure S1B-1. Intra-rater variability is observed when an image is labeled by the same rater at one week intervals.

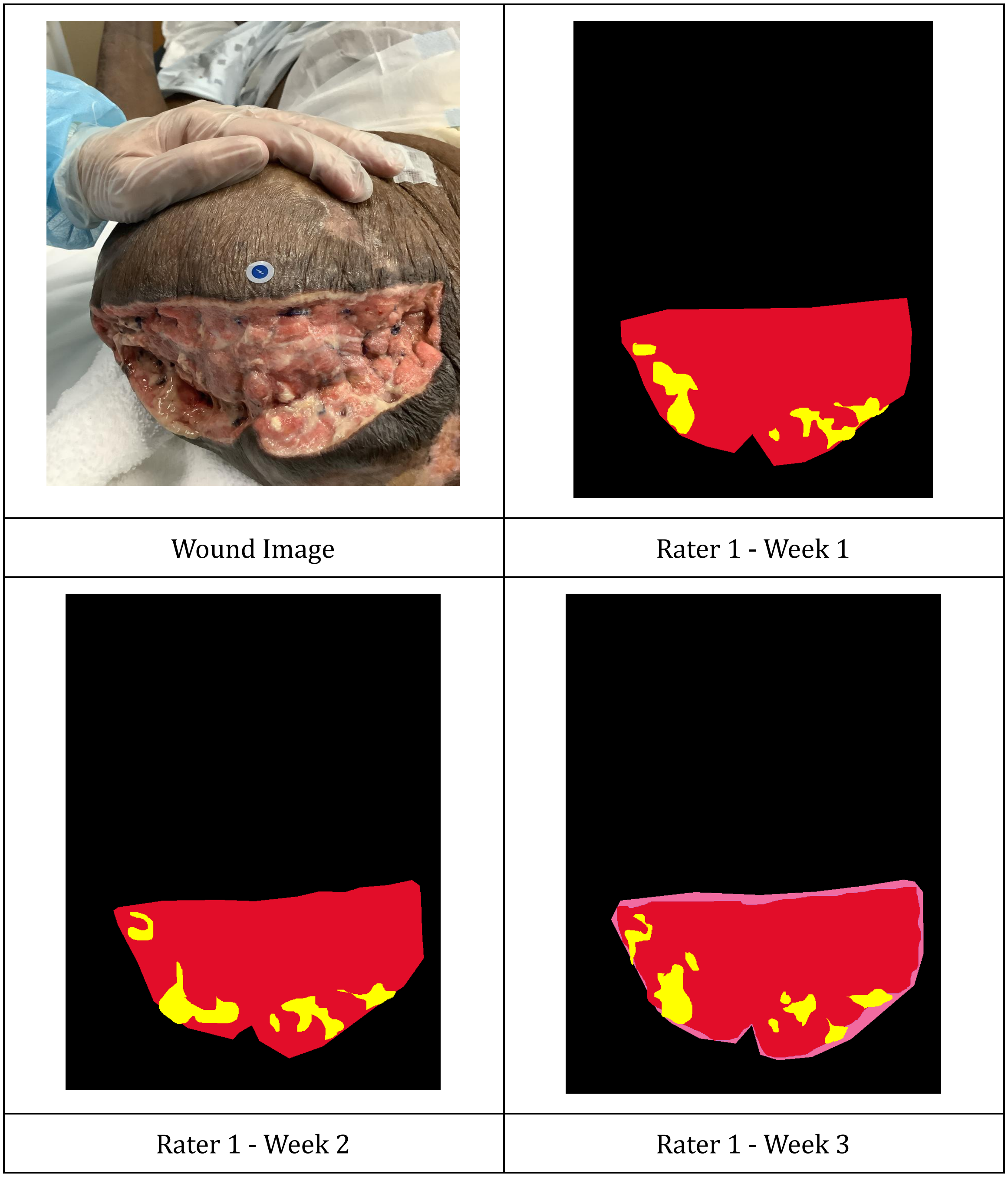

Figure S1B-2. Example 2 of Intra-rater variability which is observed when an image is labeled by the same rater at one week intervals.

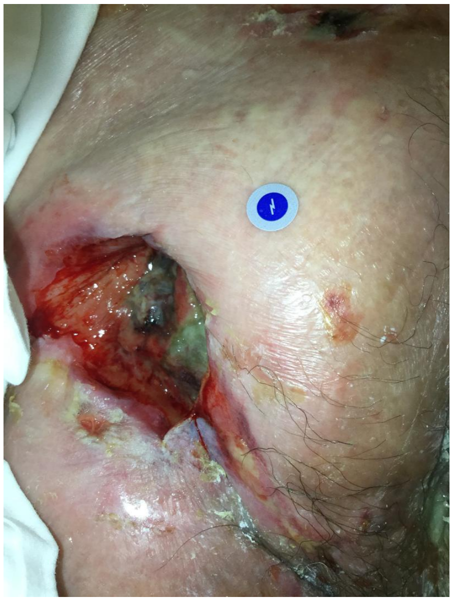	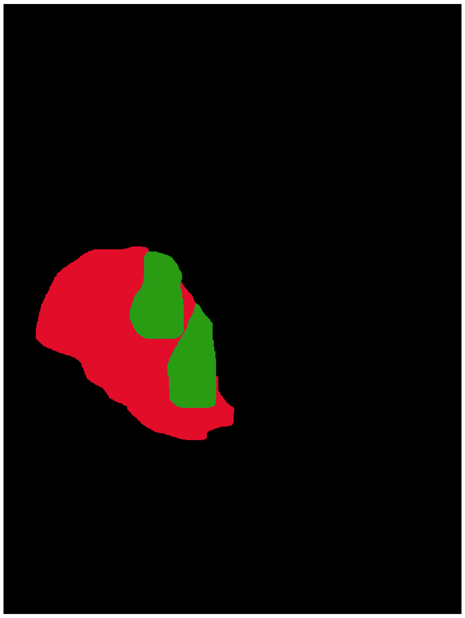
Wound Image	Rater 3 - Week 1
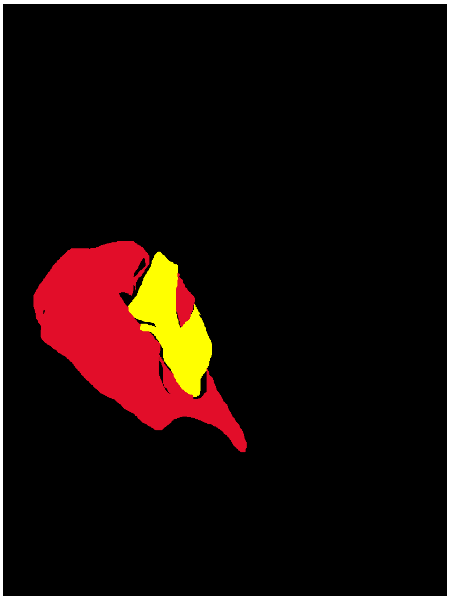	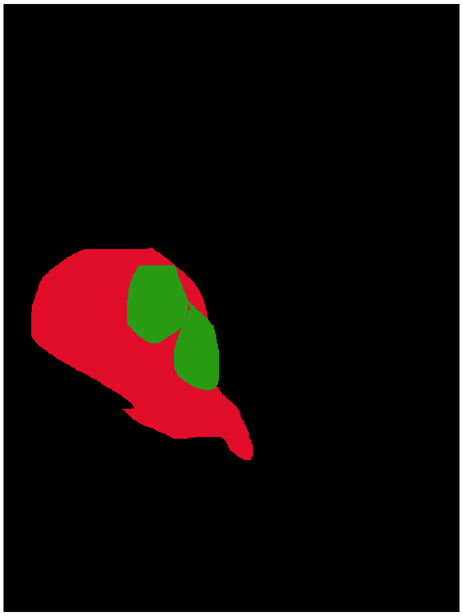
Rater 3 - Week 2	Rater 3 - Week 3

Appendix 1C

Figure S1C-1. Additional examples of predictions made using our approach.

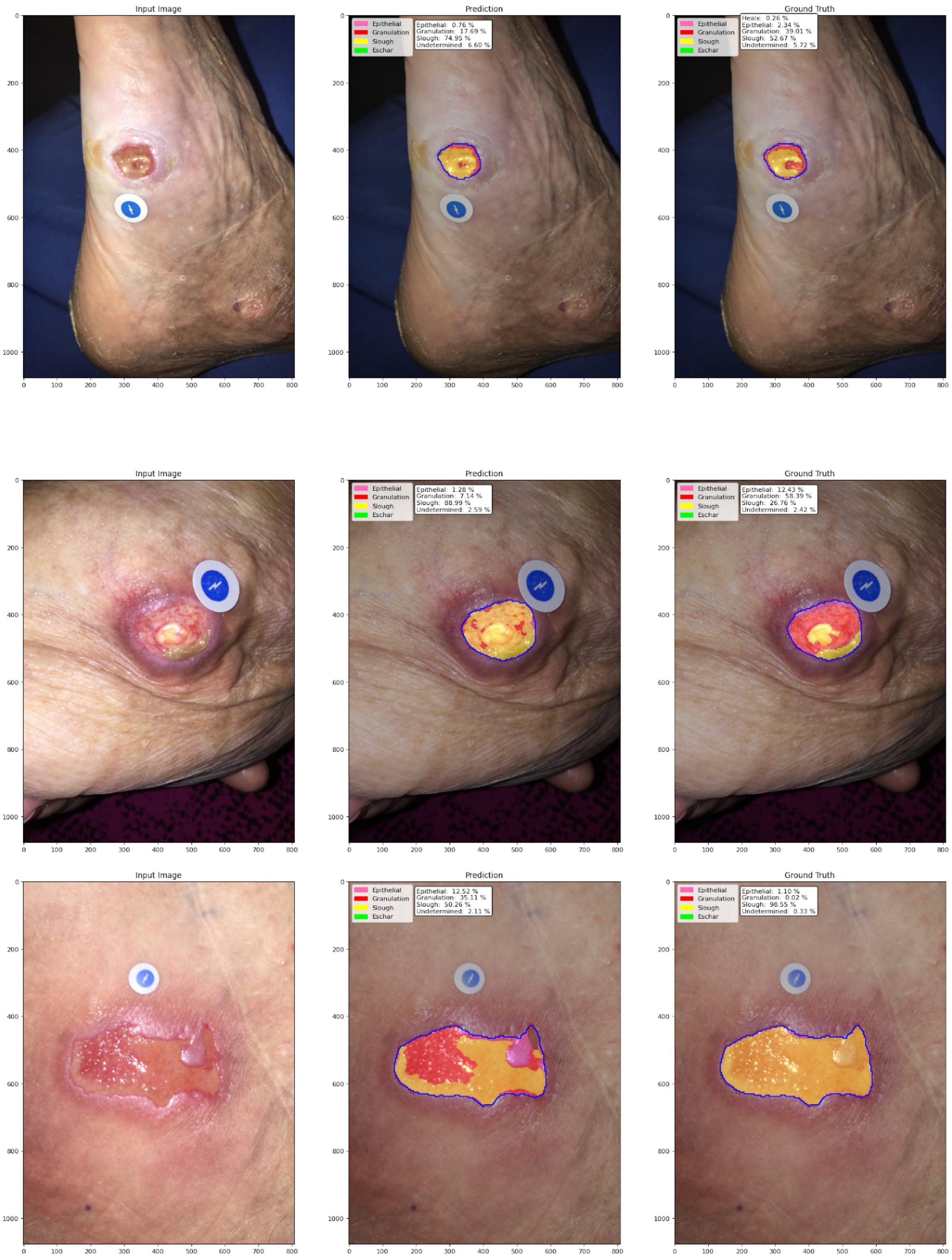

Supplement: Multimedia Appendix 1 [file mhealth_v10i4e36977_app1.pdf]
