# Supplementary material for: Fully Automated Wound Tissue Segmentation Using Deep Learning on Mobile Devices: Cohort Study
Source: JMIR Mhealth Uhealth. 2022 Apr 22;10(4):e36977. doi: 10.2196/36977 (PMC9077502; doi:10.2196/36977)

Appendix - 2A

Figure S2A-1. A screenshot of the mobile application running the deep learning models on the device. Predicted tissues are shown as overlays when using the app.

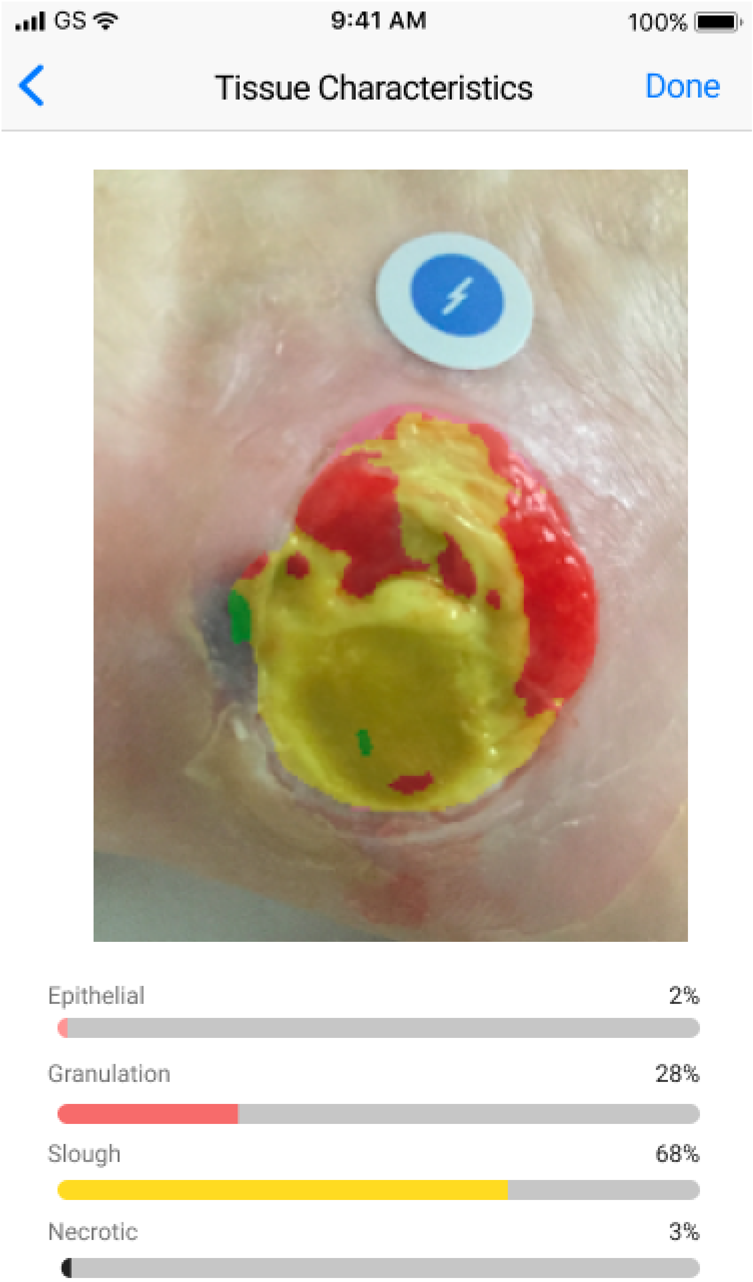

Supplement: Multimedia Appendix 2 [file mhealth_v10i4e36977_app2.pdf]
